# Enterobius vermicularis as a cause of acute appendicitis: a case report

**DOI:** 10.3389/fmed.2026.1818165

**Published:** 2026-04-15

**Authors:** Francesca Carraturo, Roberta Colicchio, Claudia Di Mento, Leonardo Continisio, Annalisa Chiodi, Maria Escolino, Maria D’Armiento, Ciro Esposito, Paola Salvatore, Mariateresa Vitiello

**Affiliations:** 1 Division of Pediatric Surgery, Federico II University Hospital, Naples, Italy; 2 Department of Molecular Medicine and Medical Biotechnology, University of Naples “Federico II,” Naples, Italy; 3 National Ph.D. Programme in One Health Approaches to Infectious Diseases and Life Science Research, Department of Public Health, Experimental and Forensic Medicine, University of Pavia, Pavia, Italy; 4 Department of Public Health, “Federico II” University of Naples, Naples, Italy; 5 CEINGE-Advanced Biotechnologies Franco Salvatore s.c.ar.l., Naples, Italy

**Keywords:** acute appendicitis, enterobiasis, *Enterobius vermicularis*, laparoscopic appendectomy, parasitological diagnosis

## Abstract

Enterobius vermicularis, commonly known as the pinworm, is a prevalent intestinal nematode, particularly affecting children worldwide. Although infection is often asymptomatic, it may occasionally lead to complications, including appendiceal involvement. The role of *E. vermicularis* in the pathogenesis of acute appendicitis remains controversial and is frequently overlooked. We report a case of a 10-year-old child who presented with intermittent right lower quadrant abdominal pain without fever or systemic symptoms. Initial ultrasound revealed small mesenteric lymph nodes, with no clear evidence of appendiceal pathology. Empirical treatment with antibiotics and anti-inflammatory agents failed to relieve the symptoms. A follow-up ultrasound demonstrated a thickened retrocecal appendix with hypoechoic intraluminal content. Due to persistent pain, a laparoscopic appendectomy was performed, revealing a white, non-segmented roundworm within the appendiceal lumen. Histopathological examination and direct microscopic analysis confirmed follicular appendicitis with intraluminal nematodes morphologically consistent with *E. vermicularis*. Postoperative parasitological examination further supported the diagnosis, with detection of characteristic eggs using the Scotch tape test, while stool examinations remained negative. These findings suggested *E. vermicularis* as the most likely etiological agent and supported its pathogenic role in the patient’s clinical presentation. The patient was treated with albendazole, followed by a second dose 2 weeks later. Simultaneous treatment was administered to household contacts, and strict hygiene measures were recommended to prevent reinfection. Clinical recovery was complete, with no postoperative complications. This case highlights the importance of considering parasitic infections in the differential diagnosis of acute abdominal pain, even in the absence of systemic inflammatory markers, particularly in endemic or underrecognized settings. Early recognition and targeted therapy are essential to prevent recurrence and intra-familial transmission.

## 1 Introduction

*Enterobius vermicularis* is one of the most prevalent intestinal helminth infection, particularly in pediatric populations. Global estimates, reported in a recent meta-analysis, indicate that 12.9% of children around the world are infected with *E. vermicularis*, with the highest rates observed in regions with inadequate hygiene and sanitation (1).

Transmission of *E. vermicularis* occurs primarily via the fecal-oral route, with infective eggs acquired through contaminated hands, fomites, or, less commonly, inhalation followed by swallowing. After ingestion, larvae hatch in the small intestine and migrate to the colon, particularly the cecum and appendix, where they mature. Gravid females then migrate nocturnally to the perianal region to deposit eggs, which become infective within a few hours and facilitate both autoinfection and person-to-person spread (2). In pediatric populations, infection is strongly associated with poor hand hygiene, overcrowded living conditions, limited access to sanitation, and communal environments such as schools and daycare centers. Intrafamilial transmission is common, and asymptomatic household carriers often contribute to persistent reinfection (2).

Although *E. vermicularis* infection is generally considered benign and self-limiting, often asymptomatic or associated with non-specific symptoms such as perianal pruritus or irritability, it can occasionally lead to more serious clinical complications. Appendiceal involvement, while relatively uncommon, has been increasingly reported (3–6) and is thought to result from several potential mechanisms, including mechanical obstruction of the appendiceal lumen by adult worms or ova, mucosal irritation, and activation of local immune response (7, 8). In some cases, the parasite may represent an incidental finding; in others, it may directly contribute to the inflammatory cascade leading to acute appendicitis (9).

The presence of *E. vermicularis* in appendectomy specimens, particularly in pediatric patients presenting with abdominal pain, should therefore be interpreted with careful integration of both clinical and histopathological findings.

Reported rates of association between *E. vermicularis* infestation and acute appendicitis vary widely, ranging from 0.2% to 41.8% among patients undergoing appendectomy for suspected appendicitis (10). This marked heterogeneity likely reflects geographic, socioeconomic, and methodological differences, and underscores the need for region-specific epidemiological data.

In Italy, evidence on the role of *E. vermicularis* in appendiceal pathology remains limited and often restricted to isolated case reports (11–13). This paucity of epidemiological data makes it difficult to establish the true burden of *E. vermicularis*–related appendiceal disease in Italy and to assess its clinical relevance. Understanding the relationship between *E. vermicularis* infection and appendicitis is essential not only for accurate diagnosis and appropriate management, but also for elucidating alternative mechanisms underlying appendiceal pathology.

In this context, the documentation of new cases is essential to expand current knowledge and better define the spectrum of clinical manifestations.

Herein, we describe a possible relationship between *E. vermicularis* and acute appendicitis in a pediatric patient, based on clinical, histopathological, and parasitological findings.

In our case, the identification of *E. vermicularis* within the appendiceal lumen, together with histological evidence of inflammation, supports a potential causative role of the parasite in the pathogenesis of appendicitis. This observation highlights the importance of considering helminthic infection in the differential diagnosis of acute abdominal pain in pediatric patients, particularly in endemic or underrecognized settings.

## 2 Case report

### 2.1 Clinical presentation

A 10-year-old child, with a history of intermittent abdominal pain localized to the right iliac fossa and no significant past medical or surgical history, presented to the emergency department of Santobono Hospital (Naples, Italy) on 8 February 2024. He reported no history of fever, vomiting, or other systemic symptoms.

Abdominal ultrasound revealed a cluster of small, subcentimetric mesenteric lymph nodes in the right flank, with no additional pathological findings. The patient was discharged with symptomatic management.

On 22 February 2024, due to persistent abdominal pain, he underwent a gastroenterology day-hospital evaluation at “Federico II” University Hospital. Blood tests were within normal limits, and an additional abdominal ultrasound did not reveal new findings. Empirical therapy was initiated, consisting of amoxicillin-clavulanate, ibuprofen, and *Lactobacillus rhamnosus* GG.

Despite treatment, the patient returned to the emergency department on 25 February 2024, with persistent right lower quadrant pain. A repeat abdominal ultrasound showed a retrocecal appendix with medial orientation and irregular caliber, with mild wall thickening of the proximal (6.3 mm) and mid-distal segments (7 mm), associated with hypoechoic intraluminal content. Color Doppler imaging did not demonstrate significant hypervascularity. Mild thickening of the surrounding mesenteric fat and clustered lymph nodes measuring up to 14 mm were observed. No free intraperitoneal fluid was detected, and the remaining abdominal structures were unremarkable.

The patient was admitted and treated with intravenous antibiotics, ceftazidime and metronidazole, from 25 February to 28 February 2024, followed by oral amoxicillin/clavulanic acid associated with ibuprofen.

**FIGURE 1:**
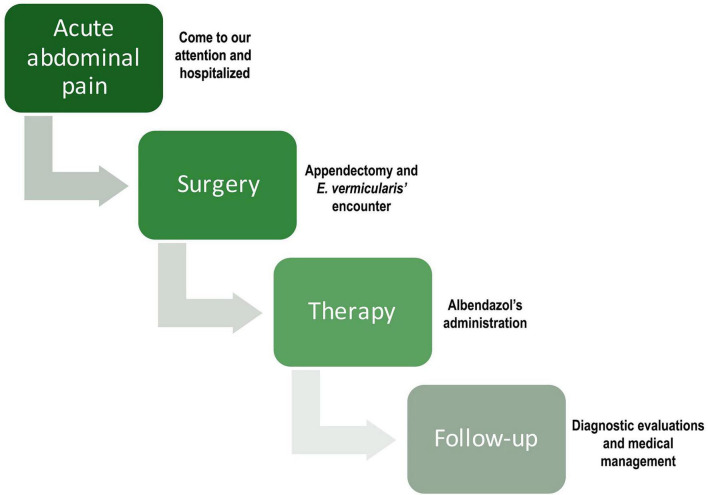
Timeline of the patient’s clinical course.

Due to persistent abdominal pain, he was subsequently transferred to the Pediatric Surgery Unit at “Federico II” University Hospital. On 1 March 2024, laparoscopic appendectomy with lysis of epiploic adhesions was performed. During dissection of the appendiceal base, a white, non-segmented, roundworm measuring a few millimeters in length was identified within the appendiceal lumen, macroscopically consistent with a helminth. Clinical recovery was complete, with no postoperative complications. As surgical post-operative controls, one control after 1 week and another after 1 month from surgery to assess the scars’ state. A brief timeline summarizing the clinical course is provided in Figure 1.

### 2.2 Diagnostic approach, differential assessment, and treatment strategy

On physical examination, the patient exhibited focal tenderness on deep palpation at McBurney’s point and a positive psoas sign, both suggestive of localized peritoneal irritation in the right lower quadrant. Although Blumberg’s sign, Rovsing’s maneuver, and the heel drop test were negative at the time of evaluation, the clinical findings, among the persistence of right iliac fossa pain on palpation, were considered consistent with an early or atypical presentation of acute appendicitis, warranting further diagnostic investigation and subsequent surgical management.

During hospitalization at the Pediatric Surgery Unit, the patient underwent hematological and biochemical laboratory tests, the results of which are summarized in Table 1.

**TABLE 1:** Results of patient’s laboratory tests.

Microbiological/parasitological tests	Normal value	Results
Stool examination for helminth eggs	Absent	Absent
Stool examination for protozoan cysts	Absent	Absent
Pinworm (*Enterobius*) detection (scotch tape test)	Absent	Microscopic examination revealed characteristic eggs of *Enterobius vermicularis*
**Complete blood count**	**Normal value**	**Results**
White blood cells	4.50–11.0 × 10^3^/μL	6.85 × 10^3^/μL
Red blood cells	4.50–5.90 × 106/μL	4.56 × 106/μL
Hemoglobin	13.0–17.0 g/dL	12.7 g/dL
Platelets	150–450 × 10^3^/μL	225 × 10^3^/μL
Neutrophils	1.80–7.0 × 10^3^/μL	3.64 × 10^3^/μL
Lymphocytes	1.0–4.8 × 10^3^/μL	2.47 × 10^3^/μL
Monocytes	0.1–0.8 × 10^3^/μL	0.59 × 10^3^/μL
Eosinophils	0.0–0.45 × 10^3^/μL	0.11 × 10^3^/μL
Basophils	0.0–0.20 × 10^3^/μL	0.04 × 10^3^/μL
Reticulocytes %	0.5–2.0%	1.41%
**Blood chemistry**	**Normal value**	**Results**
Sodium	139–146 mmol/L	141 mmol/L
Potassium	3.40–4.70 mmol/L	4.30 mmol/L
Calcium	8.80–10.80 mg/dL	9.30 mg/dL
Phosphorus	2.30–4.70 mg/dL	4.40 mg/dL
Iron	60–170 μg/dL	53 μg/dL
Ferritin	30–400 ng/mL	77 ng/mL
Glucose	60–100 mg/dL	73 mg/dL
Creatinine	0.67–1.17 mg/dL	0.54 mg/dL
Urea	15–36 mg/dL	28 mg/dL
Total protein	6.0–8.0 g/dL	5.6 g/dL
Albumin	3.80–5.40 g/dL	4.30 g/dL
Uric acid	3.50–7.20 mg/dL	2.40 mg/dL
Total bilirubin	0.15–1.20 mg/dL	0.37 mg/dL
Direct bilirubin	<0.30 mg/dL	0.19 mg/dL
Total cholesterol	<190 mg/dL	118 mg/dL
Low-density lipoprotein cholesterol	<115 mg/dL	45 mg/dL
High-density lipoprotein cholesterol	>40 mg/dL	59 mg/dL
Triglycerides	<150 mg/dL	47 mg/dL
Aspartate aminotransferase	10–50 U/L	24 U/L
Alanine aminotransferase	10–50 U/L	10 U/L
Gamma-glutamyl transferase	10–71 U/L	9 U/L
Alkaline phosphatase	129–417 U/L	147 U/L
Lactate dehydrogenase	157–272 U/L	229 U/L
Creatine phosphokinase	30–200 U/L	97 U/L
Amylase	25–101 U/L	59 U/L
Pancreatic amylase	8–51 U/L	14 U/L
Lipase	8–78 U/L	19 U/L
Cholinesterase	5,320–12,920 U/L	6,457 U/L
**Inflammatory markers/coagulation**	**Normal value**	**Result**
**Microbiological/parasitological tests**	**Normal value**	**Result**
C-reactive protein (CRP)	0–0.50 mg/dL	<0.33 mg/dL
Fibrinogen	160–350 mg/dL	214 mg/dL
Prothrombin time international normalized ratio	0.8–1.20 INR	1.14 INR
Activated partial thromboplastin time ratio	0.8–1.20 ratio	1.01 ratio
**Urine examination**	**Normal value**	**Result**
Color	–	Straw yellow
Appearance	–	Clear
pH	5.5–7.0	5.5
Specific gravity	1,005–1,030	1029
Proteins	Absent	Absent
Glucose	Absent	Absent
Ketones	Negative	Negative
Hemoglobin	Absent	Absent
Bilirubin	Absent	Absent
Urobilinogen	<1.0 mg/dL	0.2 mg/dL
Leukocyte esterase	Absent	Absent
Nitrites	Absent	Absent
Erythrocytes (cytofluorimetry)	0–14 n/μL	22 n/μL
Leukocytes	0–18 n/μL	1 n/μL
Bacteria	0–1,000 n/μL	109 n/μL
Squamous epithelial cells	0–20 n/μL	1 n/μL
**Immunological/Gastroenterological tests**	**Normal value**	**Results**
Anti-transglutaminase IgA antibodies	<9 UA: negative; 9–16 UA: doubtful; >16 UA: positive	1.0 UA (negative)
Fecal calprotectin	<50 mg/kg: normal; 50–120 mg/kg: borderline; >120 mg/kg: positive	22 mg/kg (normal)

The complete blood count revealed a white blood cell count of 6.85 × 10^3^/μL (reference range: 4.8–10.8 × 10^3^/μL), hemoglobin 12.7 g/dL (reference range: 13–17 g/dL), and platelets 225 × 10^3^/μL (reference range: 150–450 × 10^3^/μL). The differential white blood cell count was within normal limits, with no evidence of significant eosinophilia. Blood chemistry tests demonstrated normal renal and hepatic functions, with electrolytes levels within reference ranges. A mild reduction in serum iron (43 μg/dL; reference range: 50–160 μg/dL) and total protein levels (5.6 g/dL; reference range: 6.0–8.0 g/dL), was observed, in the presence of normal ferritin (77 ng/mL; reference range: 20–300 ng/mL) and albumin levels (4.3 g/dL; reference range: 3.8–5.4 g/dL). These findings were considered non-specific and did not suggest an overt systemic inflammatory or nutritional disorder. Inflammatory markers, including C-reactive protein (CRP) and fibrinogen, were within normal limits.

Physical and chemical urinalysis revealed no significant abnormalities. Mild microscopic hematuria was detected (22 erythrocytes/μL; reference range: 0–14/μL); however, its clinical relevance remained uncertain in the absence of urinary symptoms or laboratory evidence of urinary tract infection.

Given the diagnostic uncertainty and clinical suspicion of appendiceal involvement, the differential diagnoses included mesenteric lymphadenitis, early acute appendicitis, and parasitic infestation of the appendix. Mesenteric lymphadenitis was considered less likely due to the absence of systemic viral symptoms, while urinary tract infection was excluded based on the lack of pyuria or bacteriuria.

**FIGURE 2:**
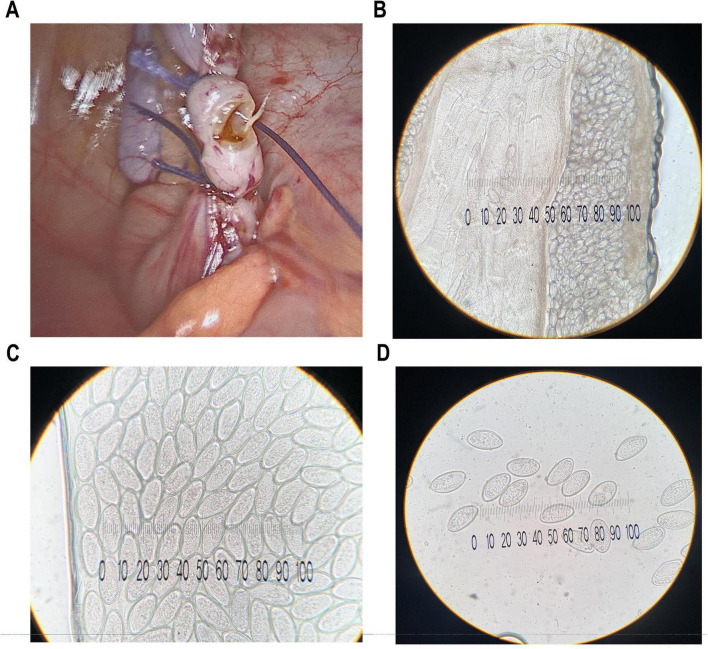
Evidence of *E. vermicularis*-associated appendiceal disease. **(A)** Intraoperative finding during laparoscopic appendectomy of a white, non-segmented roundworm in the appendiceal lumen; Longitudinal section of a gravid female, with uterine tubes filled with developing eggs, observed at **(B)** × 20 and **(C)** × 40 magnification; **(D)** Perianal Scotch tape test showing characteristic *E. vermicularis* eggs on microscopic examination (× 40 magnification).

Although an additional preoperative abdominal ultrasound might have improved diagnostic accuracy, laparoscopy exploration was indicated due to persistent localized pain and the inability to reliably exclude appendicitis on clinical grounds. During the laparoscopic procedure, at the time of transection of the appendiceal base, a white, non-segmented, round, curved parasite was observed emerging from the appendiceal lumen (Figure 2A). The appendix was subsequently excised and submitted for histopathological examination. Gross evaluation revealed inflammatory changes suggestive of a parasitic etiology. Microscopic histopathological analysis demonstrated follicular appendicitis, characterized by marked hyperplasia of mucosal and submucosal lymphoid follicles. The appendiceal lumen contained structures morphologically consistent with the cytoskeletal elements of a parasitic organism, most likely *E. vermicularis*.

**FIGURE 3:**
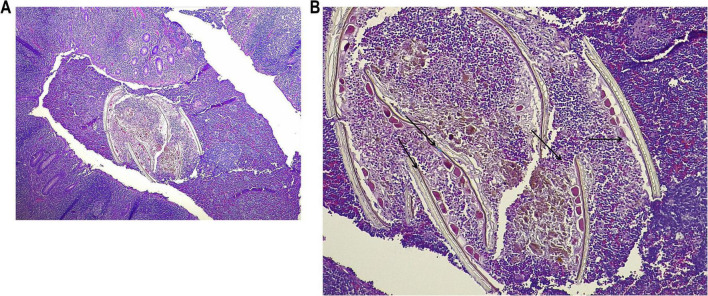
Histopathological findings of *E. vermicularis* in the Appendix (H&E stain). Histological sections of the Appendix (H&E stain) showing intraluminal parasitic structures morphologically consistent with *E. vermicularis* associated with significant inflammatory signs. Transverse and longitudinal sections of pinworms are indicated by black arrows. **(A)** × 100 magnification, **(B)** × 200 magnification.

As shown in Figure 3, transverse and longitudinal sections of the pinworm were identified and readily distinguishable by the characteristic contrast between the cuticle and internal structures on hematoxylin–eosin (H&E) staining. This staining technique clearly delineates the parasite’s morphology within the host tissue, thereby facilitating histopathological diagnosis. Parasitological direct microscopic examination of the white, non-segmented worm further confirmed the diagnosis of acute appendicitis associated with *E. vermicularis* infestation (Figures 2B,C).

Overall, these findings supported the diagnosis of parasitic appendicitis and guided subsequent therapeutic and diagnostic managements.

Clinical suspicion of parasitic infection was subsequently investigated through an extended parasitological workup, including stool examinations performed on three separate days, a perianal Scotch tape test, and serological testing for *Strongyloides stercoralis* and *Taenia solium* (IgG antibodies), as these parasites have been reported among potential causes of appendiceal involvement (14, 15).

Parasitological stool examinations were performed on three separate days between 20 February and 4 March 2024, in order to increase diagnostic sensitivity and to investigate the presence of helminth eggs and protozoan cysts. Each specimen was processed using the Para-Pak^®^ CON-Trate^®^ Stool Concentration System (Meridian Bioscience^®^), which concentrates parasitic elements through filtration and centrifugation, in accordance with the manufacturer’s instructions. All three examinations yielded consistently negative results for helminth eggs and protozoan cysts. However, stool microscopy is known to have limited sensitivity and diagnostic utility in *E. vermicularis* infections, as the ova are typically deposited in the perianal region rather than excreted via the intestinal lumen in feces.

A morning perianal Scotch tape test was therefore performed by applying a strip of transparent adhesive cellulose tape to the perianal skin in the early morning, prior to defecation or washing, to collect potentially deposited *E. vermicularis* eggs. The tape was subsequently mounted on a glass slide and examined microscopically, allowing detection of characteristic pinworm eggs and confirming the diagnosis (Figure 2D).

Serological testing revealed negative results for both anti-*S. stercoralis* antibodies, detected using an ELISA method (NovaLisa^®^ *Strongyloides* Antibody ELISA, Novatec Immunodiagnostic^®^ GmbH; diagnostic specificity 94.12% and sensitivity 89.47%), and anti-*T. solium* IgG antibodies, assessed using the NovaLisa^®^ *T. solium* IgG ELISA (Novatec Immunodiagnostic^®^ GmbH).

The patient received antiparasitic therapy with albendazole (400 mg orally as a single dose, administered with food) followed by a second dose 2 weeks later, and family members were treated at the same time. In addition, hygiene measures were recommended to prevent reinfection, including regular handwashing, laundering of linens and clothing at high temperatures, and thorough cleaning of household surfaces.

## 3 Discussion

The presence of *E. vermicularis* within the human appendix has been widely reported in histopathological studies (16, 17); however, its pathogenic role in appendiceal disease remains controversial.

While some authors interpret its detection as an incidental finding, particularly in cases lacking neutrophilic infiltration or other defining features of acute appendicitis, others have proposed that *E. vermicularis* may contribute to appendiceal pathology through a range of processes, including lymphoid hyperplasia, luminal obstruction, or low-grade inflammatory responses that do not necessarily fulfill conventional histological criteria for acute appendicitis (2–4, 7, 18). Importantly, these two interpretations are not mutually exclusive, and *E. vermicularis* may act either as an incidental colonizer or as a contributing factor, depending on host and local tissue responses.

Recent evidence further supports this dual role, emphasizing that parasitic appendicitis represents a heterogeneous entity in which helminths may act as true pathogens or incidental findings depending on the interplay between parasite burden, host response, and luminal obstruction (19).

In the present case, the patient’s initial clinical picture was non-specific, with localized right iliac fossa pain in the absence of fever and normal laboratory findings, including no leukocytosis, eosinophilia, or elevation of inflammatory markers. This presentation did not immediately suggest a parasitic etiology and is consistent with previous reports indicating that appendiceal involvement by helminths may elicit predominantly localized immune responses without significant systemic inflammation (10, 20). These features underscore an important diagnostic challenge, as routine laboratory investigations may fail to identify parasitic appendiceal disease.

An additional consideration is whether host immune factors contribute to this atypical presentation. Although our patient had no clinical history of immunodeficiency and no extended immunological testing was performed, individual variations in immune response may influence systemic inflammation. For example, selective IgA deficiency or complement pathway alterations (e.g., C3, C4) could modulate mucosal immunity and attenuate inflammatory markers, given the role of secretory IgA in intestinal defense and parasite control (21, 22). While this cannot be confirmed here, it may help explain variability in inflammatory profiles in *E. vermicularis*-associated appendiceal involvement. Further studies should investigate the impact of host immune variability, particularly mucosal and complement responses, in cases lacking typical inflammatory markers.

Furthermore, imaging findings on serial abdominal ultrasonography, namely appendiceal wall thickening, hypoechoic intraluminal content, and mesenteric lymphadenopathy, were suggestive of early appendicitis but lacked specificity for a parasitic cause. This further underscores that standard imaging modalities, while useful in identifying appendiceal involvement, are insufficient to discriminate between conventional appendicitis and parasitic-associated pathology.

Despite empirical antibiotic therapy, persistent pain prompted surgical intervention. Definitive diagnosis was established intraoperatively with the direct visualization of a white, non-segmented roundworm consistent with *E. vermicularis* protruding from the appendiceal lumen. Parasitological examination confirmed a gravid female with uterine tubes filled out by developing eggs, and histopathological analysis demonstrated intraluminal nematodes with characteristic morphological features, including a well-defined cuticle and internal structures, accompanied by prominent lymphoid hyperplasia.

The relative absence of significant purulent inflammation aligns with existing literature describing *E. vermicularis*-associated appendiceal disease as frequently characterized by minimal mucosal destruction and variable inflammatory changes rather than classic suppurative appendicitis (7–9).

Postoperative stool examinations were repeatedly negative, a finding that is well-explained by the parasite’s life cycle, as egg deposition occurs predominantly in the perianal region rather than in the intestinal lumen. Accordingly, the perianal Scotch tape test, rather than stool microscopy, remains the diagnostic method of choice and confirmed the diagnosis in this patient. This case therefore illustrates the limitations of conventional parasitological testing and reinforces the need for targeted diagnostic approaches when *E. vermicularis* infection is suspected.

The exclusion of other helminthic infections, performed as part of a broader differential diagnostic workup, including *S. stercoralis* and *T. solium*, further supported the specificity of the findings.

The patient’s rapid clinical improvement following antihelminthic therapy suggests the relevance of parasitological assessment in similar clinical scenarios.

Although *E. vermicularis* is not considered typically endemic in industrialized countries, its prevalence remains substantial due to the ease of fecal-oral transmission. This underscores an often-overlooked epidemiological reality: pinworm infections are frequently underdiagnosed due to their non-specific symptoms and the social stigma associated with parasitic diseases. The absence of perianal symptoms, such as pruritus, in this patient highlights the variability in clinical manifestations of *E. vermicularis* infection. Abdominal pain may therefore represent the sole clinical manifestation, increasing the risk of misdiagnosis or diagnostic delay, as more common gastrointestinal etiologies. Overall, this report highlights that the diagnosis of *E. vermicularis*, associated appendiceal disease requires a high index of clinical suspicion and the use of targeted diagnostic tests, even when imaging findings are non-specific, and laboratory parameters are unremarkable, particularly in pediatric populations and in communities where group-based activities facilitate transmission. It is important to acknowledge that, as a single case report, establishing a definitive causal relationship between *E. vermicularis* and appendiceal inflammation is challenging, and broader studies are needed to better clarify the potential pathogenetic role of this parasite.

Increased awareness of this diagnostic complexity is essential to avoid underrecognition, ensure appropriate therapy, and prevent reinfection through timely treatment of close contacts.

## 4 Conclusion

This case highlights that *E. vermicularis*-associated appendiceal disease represents a diagnostic challenge, as it may occur in the absence of systemic inflammatory markers and with non-specific imaging findings. It provides a valuable opportunity to explore the diagnostic, surgical, and therapeutic complexities of *E. vermicularis*-related appendicitis. Early identification and appropriate management, including surgical intervention (especially via a laparoscopic approach) and anthelmintic treatment, are crucial to prevent complications and recurrences. Moreover, this case emphasizes the importance of coordinated multidisciplinary collaboration among pediatricians, surgeons, parasitologists, and infectious disease specalists, to optimize outcomes in these uncommon but clinically significant presentations.

## Data Availability

The original contributions presented in this study are included in this article/supplementary materials, further inquiries can be directed to the corresponding authors.
